# Intergenerational continuity of loneliness and potential mechanisms: Young Finns Multigenerational Study

**DOI:** 10.1038/s41598-024-56147-6

**Published:** 2024-03-05

**Authors:** Marko Elovainio, Kaisla Komulainen, Christian Hakulinen, Katja Pahkala, Suvi Rovio, Nina Hutri, Olli T. Raitakari, Laura Pulkki-Råback

**Affiliations:** 1 Research Program Unit, Faculty of Medicine (Department of Psychology), University of Helsinki, Haartmaninkatu 3, P.O.Box 63, 00014 Helsinki, Finland; 2 Finnish Institute for Health and Welfare, Mannerheimintie 166, 00300 Helsinki, Finland; 3 Department of Public Health, University of Turku and Turku University Hospital, Kiinamyllynkatu 10, 20520 Turku, Finland; 4 Centre for Population Health Research, University of Turku and Turku University Hospital, Kiinamyllynkatu 10, 20520 Turku, Finland; 5 Research Centre of Applied and Preventive Cardiovascular Medicine, University of Turku, Kiinamyllynkatu 10, 20520 Turku, Finland; 6 Department of Pediatrics, Tampere University Hospital and Faculty of Medicine and Health Technology, Tampere University, Tampere, Finland

**Keywords:** Intergenerational transmission, Loneliness, Sex effects, Young Finns Study, Psychology, Human behaviour, Risk factors

## Abstract

Evidence on the intergenerational continuity of loneliness and on potential mechanisms that connect loneliness across successive generations is limited. We examined the association between loneliness of (G0) parents (859 mothers and 570 fathers, mean age 74 years) and their children (G1) (433 sons and 558 daughters, mean age 47 years) producing 991 parent–offspring pairs and tested whether these associations were mediated through subjective socioeconomic position, temperament characteristics, cognitive performance, and depressive symptoms. Mean loneliness across parents had an independent effect on their adult children’s experienced loneliness (OR = 1.72, 95% CI 1.23–2.42). We also found a robust effect of mothers’ (OR = 1.64, 95% CI 1.17–2.29), but not of fathers’ loneliness (OR = 1.47, 95% CI 0.96–2.25) on offspring’s experienced loneliness in adulthood. The associations were partly mediated by offspring depressive (41–54%) and anxiety (29–31%) symptoms. The current findings emphasize the high interdependence of loneliness within families mediated partly by offspring’s mental health problems.

## Introduction

Intergenerational continuity or transmission refers to the transfer of individual physical characteristics, abilities, traits, or psychosocial outcomes from parents to their children. Such continuity can arise when a parental trait affects the similar trait in their children via genetic inheritance, sociocultural transmission, or through the interplay between genetics and the environment^1,2^. Numerous studies have indicated the heritability of psychopathology, and consequently, suggested the prominence of genetics and/or gene-environment correlations in the intergenerational transmission of mental disorders^3–5^. Much less is known about the intergenerational continuity in social interaction patterns, such as persistent difficulties in forming and maintaining satisfying social relationships, that often lead to feelings of loneliness^6–8^. Loneliness in turn, can have detrimental effects on a person’s physical and mental health^9–12^.

Only relatively few studies have examined the potential association between loneliness in parents and in their offspring^13^. This is surprising, given that intergenerational processes underlying loneliness may have long-lasting negative consequences for health and wellbeing among both generations. Furthermore, most previous studies on the intergenerational transmission of loneliness examined the associations between parents and their small^14–16^ or adolescent^17^ children. Thus, the question of whether loneliness in parents is associated with loneliness in their adult children has not been fully addressed. This question merits to be studied as loneliness may be very different phenomena in old age and in mid-age adulthood compared to adolescence and small children^18–21^.

While most of the previous evidence suggests an association between parental and offspring loneliness, most studies have been conducted in parents and their young or adolescent children^14–17,22^. Some studies observed the association only between mothers and offspring^14^, and others only among offspring girls^16,17^. A recent study examining the intergenerational transmission of loneliness from parents to their adult offspring showed that^13^ higher levels of loneliness among both mothers and fathers were associated with higher levels of loneliness in their adult children. However, when examining adult children who no longer shared a household with their parents, loneliness among mothers was associated with loneliness among adult children, while no corresponding relationship was found between fathers and their adult children. It is thus possible that parents’ sex modifies the intergenerational associations of loneliness. This may reflect a stronger emotional bond of a child to their mother, arising from social and cultural norms that assign primary caregiving roles to mothers. Previous research also proposes that there might be differences between father-son and mother-daughter pairs and between discordant sex pairs in the intergenerational associations of loneliness, hypothesizing that sons and daughters’ relationship with mother and father involve different interaction experiences. For instance, it has been shown that mother-daughter pairs meet more frequently and help each other more than father-son pairs^23^. Additionally, daughters may model their mother more strongly than their father, whereas sons may model their fathers more strongly^24^.

Psychological models relevant for the potential intergenerational transmission of loneliness include the social learning theory^24,25^, and the attachment theory^26^. The social learning theory suggest that parents function as role models and their children model their behaviors and emotional reactions when being constantly exposed to them. According to the attachment theory, the emotional family atmosphere affects children’s attachment bonds and appraisal of relationships over the lifespan. Parents’ satisfaction with social relations may also be a strong contributor to the overall atmosphere within a family^27^. Parents with less experienced loneliness are likely to have better means to support social relations of their children due to higher social capital and involvement, better psychological functioning^28,29^, or a more mature personality^30,31^. Thus, it seems reasonable to expect that adult children would report relatively similar levels of loneliness as their parents.

There are multiple potential mechanisms linking parental and offspring loneliness suggested by the theories presented above and by some previous studies. One mechanism is social status that may be affected by social resources provided by ones’ family during childhood and adolescence. It has been shown that low socioeconomic status and living in a socioeconomically deprived neighborhood are both associated with more frequent experiences of loneliness and social isolation^32^. Offspring temperament may also be among the potential mediators in the intergenerational transmission of social interaction patterns and relationship qualities, given that parenting is known to predict the temperament and personality of offspring^33–35^. The most prominent temperament characteristic that may affect loneliness is sociability, referring to the degree to which a child is comfortable with and approaches new people and new situations as opposed to being shy, withdrawn or reserved^36^. Children high in sociability are less likely to be lonely because they have better social skills and fewer peer problems^37^. The ‘relationship intelligence’ hypothesis suggests that social relationships also play an important role in enhancing and maintaining cognitive abilities and vice versa, although the evidence is mostly based on animal studies^38^. It has been proposed that more frequent social contacts and positive social environment also earlier in life build cognitive capacity, including better overall cognitive performance^39^, which in turn, could affect individuals' subsequent ability to establish and maintain social relationships^40^.

Recently, it has been shown that positive social relations in childhood family predict onset of depression. Furthermore, associations between depressive symptoms and loneliness have repeatedly been shown^41,42^. Furthermore, depressive symptoms have shown to be one of the links between loneliness and many severe health outcomes^10,11,43,44^. Thus, depression is potentially a strong mediator in the relationship between early exposure to parental loneliness and experienced loneliness in adulthood. Similarly, experiences of loneliness reported by mothers both before^45^ and after childbirth^46^ have also been linked to heightened symptoms of anxiety in their offspring. Anxiety symptoms have, in turn, been associated with future feelings of loneliness in young cohorts^47,48^ and in broader population samples^49^. Therefore, anxiety may function as a mediating factor explaining the association between parental loneliness and their offspring loneliness. In sum, we tested whether the multigenerational transmission of loneliness reflects (a) shared social factors, such as low socioeconomic status, (b) shared genetic factors relating to temperament or cognitive performance or (c) some type of learning or transmission across generations indicated as depressive or anxiety symptoms.

In the present study, we examined whether loneliness experienced in adulthood is intergenerationally associated between parents and offspring in the same lineage. We also tested whether the potential association of parental loneliness with offspring loneliness was mediated by offspring subjective socioeconomic position, sociability temperament, cognitive performance, depressive symptoms, or anxiety symptoms. Furthermore, we tested whether there was a stronger intergenerational transmission for father-son and mother-daughter pairs than between discordant sex pairs. We controlled for the important covariates known to be associated with loneliness, such as age, income, education, marital status, number of children, number of siblings and parental education^50–52^.

## Results

Table 1 summarizes parental and offspring characteristics by the offspring loneliness status. Of the 991 offspring participants, 284 (29%) were classified as lonely. The mean age at time of loneliness measurements in 2018 was 47 (SD 4.7) years in G1 and 74 (SD 5.7) years in G0. Lonely G1 participants were less often married or cohabiting (63% vs 82%), had fewer children, lower income, lower sociability, more depressive symptoms, and more anxiety symptoms than those that were not lonely. Bivariate associations of all included variables are reported in Stable 1.

**Table 1:** Study characteristics according to offspring (G1) loneliness-status.

	Not lonely (N = 707)	Lonely (N = 284)	p-value for difference
Age (G1) (years)			
Mean (SD)	47 (± 4.8)	47 (± 4.6)	0.322
Age (G0) (years)			
Mean (SD)	74 (± 5.7)	74 (± 5.6)	0.853
Sex			
Female	400 (56.6%)	158 (55.6%)	0.842
Male	307 (43.4%)	126 (44.4%)	
Parental education (G0)			
Mean (SD)	11 (± 3.1)	11 (± 3.4)	0.104
Missing	2 (0.3%)	2 (0.7%)	
Number of siblings (G1)			
Mean (SD)	2.3 (± 1.1)	2.4 (± 1.2)	0.238
Missing	2 (0.3%)	0 (0%)	
Marital status			
Single	126 (17.8%)	106 (37.3%)	< 0.001
Married/cohabiting	581 (82.2%)	178 (62.7%)	
Number of children			
Mean (SD)	3.1 (± 1.3)	2.8 (± 1.4)	< 0.001
Missing	6 (0.8%)	3 (1.1%)	
Education (years)			
Mean (SD)	16 (± 3.7)	16 (± 3.6)	0.722
Missing	9 (1.3%)	1 (0.4%)	
Income			
Mean (SD)	10 (± 4.9)	8.8 (± 4.5)	< 0.001
Missing	12 (1.7%)	13 (4.6%)	
Sociability-temperament			
Mean (SD)	3.3 (± 0.68)	2.9 (± 0.74)	< 0.001
Missing	3 (0.4%)	2 (0.7%)	
Depressive symptoms			
Mean (SD)	3.3 (± 4.7)	8.9 (± 8.3)	< 0.001
Missing	40 (5.7%)	24 (8.5%)	
Anxiety symptoms			
Mean (SD)	0.19 (± 0.30)	0.49 (± 0.48)	< 0.001
Missing	5 (0.7%)	2 (0.7%)	
Cognitive performance			
Mean (SD)	0.73 (± 2.3)	0.99 (± 2.4)	0.145
Missing	62 (8.8%)	31 (10.9%)	

Before imputation/complete case data.

G0, generation 0; G1, generation 1.

In analyses adjusted for age, education and income, a significant association was found between parental loneliness and offspring subjective socioeconomic status (Β = − 0.33, 95% CI − 0.63 to − 0.04), and between parental loneliness and offspring depressive symptoms (Β = 1.76, 95% CI 0.77–2.74) and between parental loneliness and offspring anxiety symptoms (Β = 0.08, 95% CI 0.03–0.14). Similar pattern of associations was found between mothers’ loneliness and offspring mediators. There were no associations between fathers’ loneliness and any of the potential mediators (Table 2). The corresponding standardized coefficients are reported in Stable 2.

**Table 2:** Associations between parental loneliness and potential mediators.

Exposure	Mediator	Β	95% CI	p-value
Mean parental loneliness	Subjective socioeconomic position	− 0.33	− 0.63 to − 0.04	0.02
	Sociability temperament	− 0.08	− 0.19 to 0.03	0.14
	Cognitive performance	0.05	− 0.13 to 0.23	0.57
	Depressive symptoms	1.76	0.77 to 2.74	0.00
	Anxiety symptoms	0.08	0.03 to 0.14	0.00
Mothers’ loneliness	Subjective socioeconomic position	− 0.34	− 0.64 to − 0.05	0.02
	Sociability temperament	− 0.09	− 0.2 to 0.01	0.09
	Cognitive performance	− 0.03	− 0.21 to 0.14	0.69
	Depressive symptoms	2.09	1.03 to 3.15	0.00
	Anxiety symptoms	0.08	0.02 to 0.14	0.01
Fathers’ loneliness	Subjective socioeconomic position	0.02	− 0.35 to 0.38	0.92
	Sociability temperament	0.03	− 0.1 to 0.17	0.64
	Cognitive performance	0.10	− 0.11 to 0.3	0.36
	Depressive symptoms	0.57	− 0.57 to 1.72	0.33
	Anxiety symptoms	0.04	− 0.02 to 0.11	0.21

Note: Adjusted for age (G0 and G1), sex (G1), education (G1) and income (G1).

*Β*, coefficient; CI, confidence intervals. Imputed data.

The associations of loneliness among parents with loneliness of their adult offspring in analyses adjusted for confounders (age, parental education, number of siblings, sex, marital status, number of children, education, and income) are presented in Table 3. Loneliness among both parents was associated with offspring loneliness (OR = 1.72 [95% CI 1.23–2.42), and a similar pattern was observed for mothers’ loneliness. Compared to those not exposed to their mothers’ loneliness, those who had lonely mothers had a higher risk being lonelily themselves (OR 1.64 [95% CI 1.17–2.29]). No association was found between loneliness among fathers and the loneliness of their adult offspring (OR 1.47 [95% CI 0.96–2.25]). Similar results were found when using the continuous loneliness measures (Stable 1) or complete case data (Stable 2). There were no interaction effects between offspring sex and parental loneliness (p = 0.357), offspring sex and mothers’ loneliness (p = 0.438) or offspring sex and fathers’ loneliness (p = 0.480) in the association with offspring loneliness (Stable 5).

**Table 3:** Associations between loneliness in parents and offspring.

	Loneliness (G1)			Loneliness (G1)			Loneliness (G1)		
	Exposure parental loneliness			Exposure mothers’ loneliness			Exposure fathers’ loneliness		
	OR^a^	95% CI^a^	p-value	OR^a^	95% CI^a^	p-value	OR^a^	95% CI^a^	p-value
Loneliness (G0)	1.72	1.23, 2.42	0.002	1.64	1.17, 2.29	0.004	1.47	0.96, 2.25	0.073
Age (G2)	0.98	0.94, 1.02	0.331	0.98	0.94, 1.03	0.492	0.99	0.94, 1.05	0.832
Age (G1)	1.00	0.97, 1.04	0.891	1.00	0.96, 1.04	0.952	1.01	0.96, 1.06	0.686
Childhood SES	1.07	1.01, 1.12	0.012	1.06	1.00, 1.12	0.038	1.12	1.05, 1.19	< 0.001
Number of siblings	1.05	0.92, 1.20	0.428	1.07	0.93, 1.23	0.338	1.08	0.89, 1.31	0.435
Sex (female)	1.24	0.91, 1.68	0.178	1.40	1.01, 1.94	0.044	1.17	0.78, 1.77	0.445
Marital status (married/cohabiting)	0.41	0.28, 0.61	< 0.001	0.43	0.29, 0.66	< 0.001	0.39	0.23, 0.67	< 0.001
Number of children	0.97	0.85, 1.12	0.699	0.95	0.82, 1.10	0.470	1.05	0.87, 1.26	0.621
Education (years)	1.00	0.95, 1.04	0.865	1.01	0.96, 1.06	0.780	0.97	0.91, 1.03	0.252
Income	0.94	0.90, 0.97	< 0.001	0.94	0.91, 0.98	0.002	0.93	0.88, 0.97	0.001
Observations	991			873			574		
R^2^ Tjur	0.10			0.10			0.11		

Adjusted for age, sex, marital status, number of children, education and income. Numbers are odds ratios (OR) and 95%confidence intervals (95% CI). Imputed data.

^a^OR, odds ratio; CI, confidence interval.

The results from analyses evaluating mediation via subjective socioeconomic position, sociability temperament, cognitive performance, and depressive symptoms, are presented in Table 4. The ORs for the total effect of parental loneliness on offspring loneliness ranged from 1.86 [95% CI 1.25–2.47] to 1.75 [95% CI 1.24–2.47]. The direct effects of the associations between parental loneliness and offspring loneliness in G1 (OR between 1.51 and 1.73) were relatively similar in all mediators but a significant and relatively large proportion of the association between parental and offspring loneliness was explained by depressive (41%) and anxiety (31%) symptoms leading also to a stronger indirect effects (OR 1.23 [95% CI 1.07–1.42] and OR 1.17 [95% CI 1.03–1.32] respectively). Depressive (54%) and anxiety (29%) symptoms mediated a large share of the association between mothers’ loneliness and offspring loneliness (Table 4).

**Table 4:** Decomposition of the association between loneliness in parents and offspring to examine the role of potential mediators.

Mediator	OR	95% CI	% mediation	OR	95% CI	% mediation	OR	95% CI	% mediation
	Exposure parental loneliness			Exposure mothers’ loneliness			Exposure fathers’ loneliness		
Subjective socioeconomic position									
Total effect	1.77	1.24–2.53		1.70	1.20–2.42		1.50	0.96–2.35	
Direct effect	1.7	1.2–2.42		1.62	1.14–2.29		1.50	0.96–2.32	
Indirect effect	1.04	0.98–1.11	0.09	1.05	0.99–1.12	0.12	1.00	0.92–1.09	0.01
Social temperament									
Total effect	1.78	1.25–2.55		1.70	1.19–2.42		1.49	0.95–2.34	
Direct effect	1.71	1.21–2.43		1.61	1.14–2.29		1.55	1.00–2.41	
Indirect effect	1.04	0.97–1.12	0.09	1.05	0.98–1.13	0.13	0.96	0.88–1.06	− 0.11
Cognitive performance									
Total effect	1.75	1.24–2.47		1.68	1.19–2.37		1.46	0.95–2.26	
Direct effect	1.73	1.23–2.44		1.67	1.19–2.35		1.41	0.92–2.18	
Indirect effect	1.01	0.98–1.04	0.03	1.00	0.98–1.03	0.01	1.03	0.98–1.09	0.11
Depressive symptoms									
Total effect	1.86	1.25–2.78		1.77	1.19–2.64		1.52	0.95–2.44	
Direct effect	1.51	1.05–2.19		1.36	0.94–1.97		1.38	0.88–2.17	
Indirect effect	1.23	1.07–1.42	0.41	1.30	1.12–1.52	0.54	1.10	0.95–1.27	0.26
Anxiety symptoms									
Total effect	1.84	1.25–2.72		1.76	1.20–2.59		1.50	0.94–2.40	
Direct effect	1.58	1.09–2.27		1.54	1.07–2.22		1.37	0.87–2.15	
Indirect effect	1.17	1.03–1.32	0.31	1.14	1.01–1.30	0.29	1.10	0.96–1.26	0.26

Sensitivity analysis was used to assess the extent to which an unmeasured confounding variable would have had to affect both parental and offspring loneliness to invalidate our conclusions about the persistence of the effect of parental loneliness or of the mediating effect of depressive symptoms. Sensitivity analysis for unmeasured confounding showed that for unmeasured confounding to nullify the results of the direct effect of parental loneliness an unmeasured confounder would require a strength of association at least 1.58 times larger than the observed effect (E-value [95% CI lower limit], relative risk, 2.48 [1.37]). For unmeasured confounding to nullify the results of the indirect effect of depressive symptoms, an unmeasured confounder would require a strength of association at least 1.42 times larger than the observed effect (E-value [95% CI lower limit], relative risk, 1.72 [1.22]) (Stable 6). Comparable figures were found for anxiety symptoms (for the direct effect E-value [95% CI lower limit], relative risk, 2.46[1.38] and indirect effect E-value [95% CI lower limit], relative risk, 1.65 [1.26]) (Stable 6).

## Discussion

There are three key findings from this multigenerational study of almost 1000 parent—offspring pairs with data on loneliness. First, we found a robust association of parental loneliness with their adult children’s loneliness. Parental loneliness was associated with 1.5–1.7 times higher odds of offspring loneliness in midlife, after controlling for age, sex, education, income, marital status or number of children. These findings are in line with previous studies reporting associations between parental and offspring loneliness^14–17,22^, although only one recent study examined the intergenerational transmission of loneliness in adult offspring^13^.

Second, our findings suggest that the intergenerational associations of loneliness may be specific to parent’s sex. In line with some previous evidence^14^, the sex-specific associations we observed were evident among mothers but not among fathers. This may reflect the social and cultural norms that assign primary caregiving roles to mothers, in line with the attachment theory suggesting a long-lasting emotional bond between the child and the principal caregiver^53^. As a result, adult children seem to repeat the loneliness of their mothers, but not fathers. However, we did not find any interaction between parental loneliness and sex of the offspring, suggesting that the effects were similar in both male and female offspring loneliness. Furthermore, the differences in effects sizes for mother and fathers we found in this study were relatively small. Although some previous studies have suggested that the associations of parents’ loneliness with offspring loneliness may differ across sons and daughters, given the potential sex differences in interaction and sex-specific role models, evidence is still largely missing^16,17,24^.

Third, we found that the association between parental and offspring loneliness was partially mediated by offspring depressive symptoms. Depressive symptoms explained 40% of the association between mean parental loneliness and offspring loneliness, and 53% of the association of mothers’ loneliness with offspring loneliness, which can be considered as relatively large shares of the total association. These proportions are comparable to findings from a recent longitudinal large-scale study reporting that mediation through smoking status explained 37% of the association between socioeconomic status and mortality^54^. In contrast to depressive symptoms, subjective socioeconomic position, sociability temperament or cognitive performance did not mediate any of the intergenerational associations of loneliness in our study.

The finding that depressive symptoms acted as a powerful mediator in association between parental and offspring loneliness is plausible. Parental loneliness may be a general indicator of poor-quality social interaction or social atmosphere in the family that may in turn affect offspring mental health, including depressive symptoms. A recent study showed that poor family relations in adolescence were associated with an increased risk of later in-patient treatment for a psychiatric diagnosis until age 36–45, even when adjusting for other adverse conditions in childhood^55^. The association between depressive symptoms and loneliness, in turn, has been shown by many independent studies^41,42^ and depressive symptoms have also mediated loneliness to multiple health outcomes^10,11,43,44^. The finding that anxiety symptoms acted as mediating factors in the transmission of loneliness from parents to offspring also aligns with previous research indicating an association between parental loneliness and decreased mental health in the offspring^46^, subsequently heightening the likelihood of these children experiencing loneliness later in life^47–49^. According to some accounts, depressive mood and experienced loneliness may be somewhat overlapping concepts, and feelings of loneliness and isolation are sometimes included in scales or screening instruments assessing depression^56^, even though loneliness is not considered a key diagnostic symptom in depression. Although it is reasonable to assume that the association between loneliness and depression is bi-directional the studies examining the temporality of the association suggest that loneliness is a stronger predictor of depression than vice versa (e.g.^57^).

None of the other tested mediators showed a significant mediation effect in the association between experienced parental and offspring loneliness. Subjective social status was, however, associated with loneliness reported by both parents combined or by mothers, suggesting that social relations in the family may affect experienced social status in adulthood, although subjective social status, in turn, does not seem to be associated with loneliness experiences of the next generation. Sociability temperament was also not a significant mediator and parental loneliness was not associated with offspring sociability temperament. This may suggest that the association between parental and offspring loneliness is not explained by genetic similarities, because temperament has been shown to be strongly determined by genes^58^. However, temperament has not shown to have a clear pattern of inheritance component^59^ and although it has been shown that various personality and temperament characteristics are associated with experienced loneliness, the findings do not strongly support the idea that people with higher sociability would be less or more lonely than others^37,60,61^. Our results do not support the idea that inherited or developed sociability temperament would be responsible for the association between parental and offspring loneliness. Similarly, the association was not mediated by offspring cognitive performance and thus no support was found for the ‘relationship intelligence’ hypothesis. It may be that the potential effects of more frequent social contacts and positive social environment on cognitive capacity and consequent ability to establish and maintain social relationships^40^ only are evident in old age, as suggested by a recent study^62^.

There are some limitations that need to be considered. As most longitudinal cohort studies, due to the selective attrition, the study participants are probably healthier and from better socioeconomic position than the general population. This could be reflected in less experienced loneliness in our study^63^. It has also been shown that analyses based on data from longitudinal cohort studies do not necessarily bias risk factor-outcome associations^64^. We only used data from one measurement phase of the Young Finns study and thus, cannot ensure the direction of the associations, although the multigenerational design makes reverse causality less obvious source of bias. To note, parents (G0) and their children (G1) responded independently of each other to the loneliness questions (and all other questions), thus they had no knowledge of what the other part of the dyad had reported. This makes shared variance minimal. Parents and children share the same social environment during the child’s growing years in the family and are, in most cases, also genetically related. Twin-studies show that genes account for about from one-third to 50% of the variation in most psychological phenomena^65^. This is not in conflict with psychological models of intergenerational transmission, even though the heritability of loneliness would suggest that a significant part of the found association is due to shared genes. Due to our study design, we are not able to disentangle environmental from shared genetic influences. However, if our findings would be only explained by the shared genetic background of parents and their offspring, we probably would have found stronger mediating effect by sociability temperament or cognitive performance, that both have strong genetic component. We also cannot rule out the potential effects of residual confounding, especially for the association between the mediator and the outcome. However, the E-value indicated that a considerable unmeasured confounding would be needed to explain away the found associations between parental and offspring loneliness^66^. There are, of course, other potential mediators that were not tested in our study. It has been suggested that the mother's prenatal depression may affect the fetus or the newborn, and so parental depression may also be a plausible mediator, but we did not have this information in our data set. Furthermore, this would have meant that maternal prenatal depression would have very long-lasting effects on offspring well-being. To test the directions of all associations included in our study, multiple data points from both generations of all measured variables would have been required. Collecting data with such designs may be worth considering in the future.

In sum, we found a robust sex-specific association between parental loneliness and their adult offspring loneliness, which was also evident between mothers and the offspring adults. Loneliness is a strong risk factor for adverse health outcomes, and thus, identifying and intervening on modifiable risk factors for loneliness is important. Our findings underline the potential importance of the family-life social experiences in the development of adult social relations, loneliness as well as depressive and anxiety symptoms. Interventions to reduce loneliness could target the whole families and their social environments to support positive and close relationships within and outside family life that could break the intergenerational chains of poor social relations.

## Methods

### Sample

Data were from the ongoing prospective Young Finns Multigenerational Study (YFMS) (http://youngfinnsstudy.utu.fi), which comprises six representative Finnish birth cohorts from childhood to adulthood. This multidisciplinary study has been conducted in five university cities with medical schools (i.e., in Helsinki, Kuopio, Oulu, Tampere, and Turku) and the regions surrounding them Raitakari et al.. The YFMS is conducted in compliance with the Helsinki Declaration and approved by the ethical committees of the Hospital District of Southwest Finland and the European Research Council. All individuals and/or legal guardians have signed a written informed consent to take part in the study.

The baseline study in 1980 included 3596 randomly selected children and adolescents aged 3–18 (G1) and their parents (G0). G0 and G1 participants were linked through their personal identification codes. The parent generation (G0, N = 2452) and the offspring generation (G1, N = 2127) answered to a psychological questionnaire, including experienced loneliness in 2018. The study sample included G1 participants who had information on loneliness available and could be connected to their parents. The final sample included 991 original participants and their parents that could be connected to their mothers or fathers (56.3% female). Mean age was for G1 47 years, and for G0 74 years at the times when loneliness was measured in 2018.

## Measures

### Loneliness

Loneliness in both the parent generation (G0) and the offspring generation (G1) was measured through self-reports in 2018 using the short version of the UCLA loneliness scale^67^, developed by Hughes and co-workers^68,69^. The scale includes three items (“I feel left out”, “I feel isolated from others”, and “I lack companionship”), to which responses options include “hardly ever”, “some of the time”, and “often” (range from 3 to 9). The three-item loneliness scale has demonstrated a good reliability and both concurrent and discriminant validity^68^. The Cronbach’s alpha in the current sample was 0.73. As in previous studies^70^, we used 5/6 points as a cut-off point for loneliness vs. not, so that those who scored at least 6 out of 9 were defined as lonely in both generations. Loneliness for both parents was calculated by first calculating the mean of both parents and then classifying that mean based on the same cut-off point.

### Mediators

Subjective socioeconomic position was measured using a self-anchoring scale in the form of a 10 rung ladder^71^. Participants were given the drawing of a ladder with the following instructions: “Think of this ladder as representing where people stand in society. At the top of the ladder are the people who are best off—those who have the most money, most education, and the best jobs. At the bottom are the people who are worst off—who have the least money, least education and the worst jobs or no job. The higher up you are on this ladder, the closer you are to people at the very top and the lower you are, the closer you are to the bottom. Where would you put yourself on the ladder? Please place a large ‘X’ on the rung where you think you stand.” Sociability temperament characteristics was self-reported by the participants using the sociability dimension of the Emotionality-Activity-Sociability Temperament Survey presented by^36^. Sociability was assessed using five items (e.g., “I like to be with people”; “I prefer working with others rather than alone”). The Cronbach alpha of the scale was 0.79. Cognitive performance was assessed using the Cambridge Neuropsychological Test Automated Battery (CANTAB, Cambridge Cognition, Cambridge, UK). The YFMS test battery included four tests assessing different domains of cognitive function: visual memory and associative new learning (Paired Associates Learning test), reaction and movement time (Reaction Time test), visual processing and sustained attention (Rapid Visual Information Processing test) and short-term working memory and executive control (Spatial Working Memory test). Each of these four tests comprised several variables. As described elsewhere^72^, principal component analysis was conducted in the complete cognitive function data, and the first component resulting from this analysis was considered an indicator of global cognitive function (greater value indicates better cognitive function). Depressive symptoms were derived from questionnaire self-reports to the 21-item Beck Depression Inventory (BDI-II)^73^, where participants rate the severity of 21 depressive symptoms experienced in the past 2 weeks on scales ranging from 0 (symptom absent) to 4 (very severe). Anxiety symptoms were measured using the brief measure for assessing generalized anxiety disorder (GAD-7)^74^. That asked patients how often, during the last 2 weeks, they were bothered by each symptom. Response options were “not at all,” “several days,” “more than half the days,” and “nearly every day,” scored as 0, 1, 2, and 3, respectively.

### Confounders

In addition to age and sex (G0 and G1), the socioeconomic status (SES) (G0 and G1) and the number of children in G1 were included as confounders in the analyses. SES was measured by personal gross income (using a 21-point scale, G1) and total years of education (G0 and G1). All potential confounders were measured in 2018.

### Statistical analyses

The associations between parental (G0) and offspring (G1) loneliness were analyzed using logistic regression models with separate models for (1) the mean of parental loneliness, (2) mothers’ loneliness and (3) fathers’ loneliness as exposures. The models were adjusted for G0 age, and G1 age, sex, marital status, number of children, education (years) and income. Missing values on all variables except for the outcome were imputed using multiple imputation with chained equations^75,76^. In total, five imputed datasets were generated using all variables included except loneliness and results were combined using Rubin’s rules.

**Figure 1:**
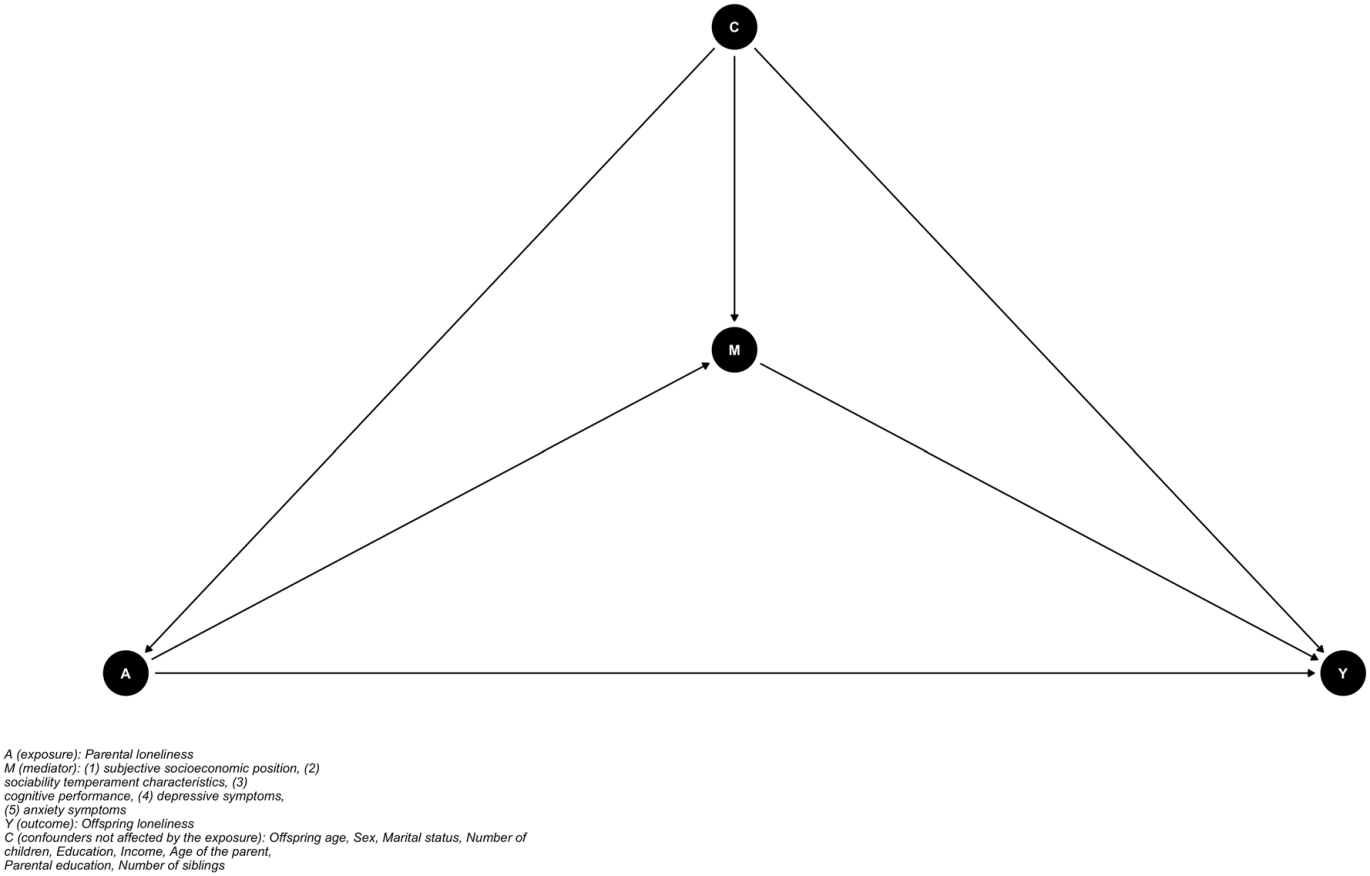
Directed Acyclic Graph (DAG) for the analysis of the contribution of (1) subjective socioeconomic position, (2) sociability temperament characteristics, (3) cognitive performance, (4) depressive symptoms and (5) anxiety symptoms to the association between parental loneliness (A) and offspring loneliness (Y).

The hypothesized associations between parental (parental, mothers’, and fathers’) loneliness (the exposure) and offspring loneliness (the outcome), with subjective socioeconomic status, sociability temperament, cognitive performance, depressive symptoms, and anxiety symptoms as mediators is shown as a Directed Acyclic Graph in Fig. 1. In this structure, subjective socioeconomic status, sociability, cognitive performance, depressive symptoms, and anxiety symptoms are hypothetical mediators. Mediation was evaluated using a regression-based counterfactual mediation approach. Separate models were conducted for all included mediators (all mediators measured in G1) and exposures (mean parental loneliness, mother’s loneliness, and fathers’ loneliness).

Interaction terms between parental loneliness and all mediators (p > 0.05) did not suggest the association between mediators and offspring loneliness to differ by parental loneliness (STable 3). Thus, we estimated the natural direct effect of parental loneliness on the offspring loneliness, the natural indirect effect that flows through each mediator, and the proportions mediated in the absence of an exposure-mediator interactions where the natural direct effect can be interpreted as the effect of parental loneliness on the outcome (change in outcome when exposure moves from 0 (not lonely) to 1 (lonely) with the mediators being distributed as in the reference group, here not lonely). The natural indirect effect describes mediation, that is the part of the association between parental and offspring loneliness due to mediators. The counterfactual effects were estimated as a combination (sums and products) of the regression coefficients obtained from:

1. A linear model for the association between the exposure (parental, mothers’, and fathers’ loneliness) and the mediators (subjective socioeconomic status, sociability temperament, cognitive performance, depressive symptoms, and anxiety symptoms), adjusted for age, sex, parent age, marital status, number of children, income, and education.
2. A logistic model for the outcome (offspring loneliness), including the exposure (parental, mothers’, and fathers’ loneliness), the mediators (separate models for each potential mediator), and confounders (age, sex, parent age, marital status, number of children, income, and education). Separate models were performed for each exposure (parental, mothers’, and fathers’ loneliness). We used delta method to compute counterfactual effect estimates and 95% Confidence Intervals (CI). All the counterfactual effects were reported on the odds ratio scale conditional on covariates. The proportion of association mediated by mediators were calculated as Direct Effect * (Indirect Effect−1)/(Total Effect−1).

All the analyses were performed with R software (version 4.0.3) and, “mice” (for multiple imputation), “intregmed”^77^ and “CMAverse”^78^ R packages.

### Supplementary Information

Supplementary Tables.

## Data Availability

Data used in the current study may be obtained from the University of Turku. Restrictions apply to the availability of these data, which were used under licence for this study. For information on accessing the data see: https://youngfinnsstudy.utu.fi.
