# Supplementary material for: Intergenerational continuity of loneliness and potential mechanisms: Young Finns Multigenerational Study
Source: Sci Rep. 2024 Mar 5;14:5465. doi: 10.1038/s41598-024-56147-6 (PMC10915156; doi:10.1038/s41598-024-56147-6)
Supplement: Supplementary file 1 — Supplementary Tables. [file 41598_2024_56147_MOESM1_ESM.docx]

Supplement: **Intergenerational continuity of loneliness and potential mechanisms - Young Finns Multigenerational Study**

Marko Elovainio, Kaisla Komulainen, Christian Hakulinen, Katja Pahkala, Suvi Rovio,

Nina Hutri, Olli T. Raitakari & Laura Pulkki - Råback

**STable 1. *Bivariate associations of all included variables (Pearson correlation coefficients, complete cases)***

**STable 2. Associations between parental loneliness and potential mediators. Abbreviations: β beta coefficient, CI confidence intervals. Imputed and standardized data.**

**STable 3. Associations between loneliness in parents and offspring. Adjusted for age, parental education, number of siblings, sex, marital status, number of children, education and income. Continuous loneliness measure and complete case analyses.**

**STable 4. Associations between loneliness in parents and offspring. Adjusted for age, parental education, number of siblings, sex, marital status, number of children, education and income. Dichotomous loneliness measure and complete cases.**

**STable 5. Interactions between parental (G0) loneliness and potential mediators.**

**STable 6. Sensitivity analysis of mediation analysis of parental and offspring loneliness.**

**STable 1. *Bivariate associations of all included variables (Pearson correlation coefficients, complete cases)***

| Variable | 1 | 2 | 3 | 4 | 5 | 6 | 7 | 8 | 9 | 10 | 11 | 12 | 13 | 14 | 15 |
| --- | --- | --- | --- | --- | --- | --- | --- | --- | --- | --- | --- | --- | --- | --- | --- |
|  |  |  |  |  |  |  |  |  |  |  |  |  |  |  |  |
| 1.Loneliness |  |  |  |  |  |  |  |  |  |  |  |  |  |  |  |
|  |  |  |  |  |  |  |  |  |  |  |  |  |  |  |  |
| 2. Parental loneliness | .11** |  |  |  |  |  |  |  |  |  |  |  |  |  |  |
|  |  |  |  |  |  |  |  |  |  |  |  |  |  |  |  |
| 3. Age (G1) | -.03 | .03 |  |  |  |  |  |  |  |  |  |  |  |  |  |
|  |  |  |  |  |  |  |  |  |  |  |  |  |  |  |  |
| 4. Age (G2) | -.01 | .05 | .67** |  |  |  |  |  |  |  |  |  |  |  |  |
|  |  |  |  |  |  |  |  |  |  |  |  |  |  |  |  |
| 5. Childhood SES | .05 | -.06* | -.19** | -.03 |  |  |  |  |  |  |  |  |  |  |  |
|  |  |  |  |  |  |  |  |  |  |  |  |  |  |  |  |
| 6. Number of siblings | .04 | .07* | .24** | .31** | -.12** |  |  |  |  |  |  |  |  |  |  |
|  |  |  |  |  |  |  |  |  |  |  |  |  |  |  |  |
| 7. Marital status (married / cohabiting) | -.04 | .00 | .06 | -.03 | .01 | -.02 |  |  |  |  |  |  |  |  |  |
|  |  |  |  |  |  |  |  |  |  |  |  |  |  |  |  |
| 8. Number of children | -.12** | -.07* | -.32** | -.22** | .09** | -.06 | -.04 |  |  |  |  |  |  |  |  |
|  |  |  |  |  |  |  |  |  |  |  |  |  |  |  |  |
| 9. Education (years) | -.01 | -.04 | -.12** | .01 | .38** | -.09** | .00 | .06 |  |  |  |  |  |  |  |
|  |  |  |  |  |  |  |  |  |  |  |  |  |  |  |  |
| 10. Income | -.13** | -.05 | -.00 | .06* | .27** | -.07* | .08* | .15** | .24** |  |  |  |  |  |  |
|  |  |  |  |  |  |  |  |  |  |  |  |  |  |  |  |
| 11. Subjective socioeconomic status | -.20** | -.06 | .02 | .10** | .18** | -.02 | .02 | .12** | .27** | .48** |  |  |  |  |  |
|  |  |  |  |  |  |  |  |  |  |  |  |  |  |  |  |
| 12. Sociability temperament | -.23** | -.05 | .00 | -.01 | -.02 | .01 | .09** | .12** | -.01 | .13** | .24** |  |  |  |  |
|  |  |  |  |  |  |  |  |  |  |  |  |  |  |  |  |
| 13. Depressive symptoms | .39** | .10** | -.01 | -.04 | -.03 | .01 | .05 | -.08* | -.05 | -.16** | -.18** | -.21** |  |  |  |
|  |  |  |  |  |  |  |  |  |  |  |  |  |  |  |  |
| 14. Anxiety symptoms | .35** | .09** | -.07* | -.09** | .01 | -.01 | .04 | -.05 | -.00 | -.12** | -.14** | -.17** | .78** |  |  |
|  |  |  |  |  |  |  |  |  |  |  |  |  |  |  |  |
| 15. Cognitive performance | .05 | .01 | -.28** | -.15** | .19** | -.08* | -.06 | .17** | .21** | .22** | .07* | -.04 | .01 | -.02 |  |
|  |  |  |  |  |  |  |  |  |  |  |  |  |  |  |  |
| 16. Sex (female) | .01 | .03 | .02 | .06 | .03 | -.01 | -.07* | .02 | -.16** | .25** | .02 | -.12** | -.14** | -.12** | .09** |
|  |  |  |  |  |  |  |  |  |  |  |  |  |  |  |  |

*Note.* * indicates *p* < .05. ** indicates *p* < .01.

| STable 2. Associations between parental loneliness and potential mediators. Abbreviations: β beta coefficient, CI confidence intervals. Imputed and standardized data. | | | | |
| --- | --- | --- | --- | --- |
| *Exposure* | *Mediator* | *β* | *95% CI* | *p-value* |
| Mean parental loneliness | Subjective socioeconomic position | -0.08 | -0.14 - -0.01 | 0.02 |
|  | Sociability temperament | -0.05 | -0.11 - 0.02 | 0.17 |
|  | Cognitive performance | 0.00 | -0.07 - 0.08 | 0.96 |
|  | Depressive symptoms | 0.11 | 0.04 - 0.17 | 0.00 |
|  | Anxiety symptoms | 0.09 | 0.03 - 0.15 | 0.01 |
| Mothers’ loneliness | Subjective socioeconomic position | -0.07 | -0.14 - 0 | 0.04 |
|  | Sociability temperament | -0.06 | -0.12 - 0.01 | 0.10 |
|  | Cognitive performance | -0.01 | -0.07 - 0.06 | 0.83 |
|  | Depressive symptoms | 0.13 | 0.06 - 0.19 | 0.00 |
|  | Anxiety symptoms | 0.08 | 0.01 - 0.15 | 0.02 |
| Fathers’ loneliness | Subjective socioeconomic position | 0.00 | -0.08 - 0.09 | 0.94 |
|  | Sociability temperament | 0.02 | -0.06 - 0.11 | 0.57 |
|  | Cognitive performance | 0.08 | 0 - 0.17 | 0.06 |
|  | Depressive symptoms | 0.03 | -0.05 - 0.11 | 0.43 |
|  | Anxiety symptoms | 0.05 | -0.04 - 0.13 | 0.27 |

Note: Adjusted for age (G0 and G1), sex (G1), education (G1) and income (G1).

| **Stable 3. Associations between loneliness in parents and offspring. Adjusted for age, sex, parental education, number of siblings, marital status, number of children, education and income. Numbers are odds ratios (OR) and 95%confidence intervals (95% CI). Continuous loneliness measure** | | | | | | | | | |
| --- | --- | --- | --- | --- | --- | --- | --- | --- | --- |
|  | Loneliness (G1) | | | Loneliness (G1) | | | Loneliness (G1) | | |
|  | Exposure parental loneliness | | | Exposure mothers‘ loneliness | | | Exposure fathers’ loneliness | | |
| *Predictors* | *Estimates* | *CI* | *p* | *Estimates* | *CI* | *p* | *Estimates* | *CI* | *p* |
| Loneliness (G0) | 0.17 | 0.10 – 0.23 | **<0.001** | 0.14 | 0.07 – 0.20 | **<0.001** | 0.10 | 0.01 – 0.18 | **0.034** |
| Age (G1) | -0.03 | -0.06 – -0.00 | **0.029** | -0.03 | -0.06 – -0.00 | **0.032** | -0.02 | -0.05 – 0.02 | 0.282 |
| Age (G0) | -0.00 | -0.03 – 0.02 | 0.763 | 0.00 | -0.02 – 0.02 | 0.945 | -0.00 | -0.03 – 0.02 | 0.786 |
| Parental education (G0) | 0.04 | 0.01 – 0.07 | **0.014** | 0.04 | 0.00 – 0.07 | **0.037** | 0.07 | 0.03 – 0.11 | **0.001** |
| Number of siblings | 0.06 | -0.03 – 0.14 | 0.178 | 0.07 | -0.01 – 0.16 | 0.102 | 0.08 | -0.05 – 0.21 | 0.223 |
| Sex (female) | -0.04 | -0.23 – 0.15 | 0.699 | 0.02 | -0.19 – 0.23 | 0.852 | -0.12 | -0.38 – 0.14 | 0.366 |
| Marital status (married / cohabiting) | 0.02 | -0.09 – 0.13 | 0.725 | 0.04 | -0.08 – 0.16 | 0.481 | 0.01 | -0.13 – 0.16 | 0.865 |
| Number of children | -0.15 | -0.22 – -0.08 | **<0.001** | -0.16 | -0.23 – -0.08 | **<0.001** | -0.13 | -0.22 – -0.03 | **0.011** |
| Education (years) | 0.00 | -0.03 – 0.03 | 0.889 | 0.00 | -0.03 – 0.03 | 0.934 | -0.01 | -0.05 – 0.02 | 0.429 |
| Income | -0.04 | -0.06 – -0.02 | **<0.001** | -0.04 | -0.06 – -0.02 | **0.001** | -0.04 | -0.07 – -0.01 | **0.003** |
| R^2^ / R^2^ adjusted | 0.078 / 0.068 | | | 0.071 / 0.059 | | | 0.067 / 0.049 | | |

| **STable 4 Associations between parents and offspring loneliness. Adjusted for age, parental education, number of siblings, sex, marital status, number of children, education and income. Numbers are odds ratios (OR) and 95%confidence intervals (95% CI). Complete case analyses.** | | | | | | | | | |
| --- | --- | --- | --- | --- | --- | --- | --- | --- | --- |
|  | **G1 loneliness** | | | **G1 loneliness** | | | **G1 loneliness** | | |
|  | *Exposure parental oneliness* | | | *Exposure mothers’ loneliness* | | | *Exposure fathers’ loneliness* | | |
| *Predictors* | *OR* | **95% CI** | *p-values* | *OR* | **95% CI** | *p-values* | *OR* | **95% CI** | *p-values* |
| G0 loneliness | 1.77 | 1.25 – 2.50 | **0.001** | 1.58 | 1.11 – 2.23 | **0.011** | 1.54 | 0.99 – 2.37 | 0.052 |
| Age (G1) | 0.98 | 0.93 – 1.02 | 0.324 | 0.98 | 0.94 – 1.03 | 0.527 | 0.99 | 0.93 – 1.05 | 0.741 |
| Age G0 | 1.00 | 0.96 – 1.04 | 0.973 | 1.00 | 0.96 – 1.04 | 0.852 | 1.01 | 0.96 – 1.06 | 0.712 |
| Parental education (G0) | 1.07 | 1.01 – 1.12 | **0.017** | 1.06 | 1.00 – 1.12 | 0.064 | 1.12 | 1.05 – 1.20 | **0.001** |
| Number of siblings | 1.03 | 0.89 – 1.18 | 0.731 | 1.05 | 0.91 – 1.22 | 0.498 | 1.02 | 0.82 – 1.26 | 0.859 |
| Sex (female) | 1.23 | 0.90 – 1.70 | 0.198 | 1.33 | 0.95 – 1.88 | 0.101 | 1.21 | 0.79 – 1.86 | 0.372 |
| Marital status (married) | 0.41 | 0.27 – 0.62 | **<0.001** | 0.39 | 0.25 – 0.61 | **<0.001** | 0.41 | 0.24 – 0.70 | **0.001** |
| Number of children | 0.98 | 0.85 – 1.13 | 0.770 | 0.97 | 0.83 – 1.13 | 0.703 | 1.03 | 0.85 – 1.25 | 0.749 |
| Education (years) | 0.99 | 0.95 – 1.04 | 0.818 | 1.00 | 0.95 – 1.05 | 0.874 | 0.97 | 0.91 – 1.03 | 0.313 |
| Income | 0.94 | 0.91 – 0.97 | **0.001** | 0.94 | 0.91 – 0.98 | **0.003** | 0.93 | 0.88 – 0.97 | **0.001** |
| Observations | 927 | | | 814 | | | 547 | | |
| R^2^ Tjur | 0.071 | | | 0.070 | | | 0.076 | | |

| **STable 5. Interactions between parental (G0) loneliness and potential mediators.** | | | |
| --- | --- | --- | --- |
| *Interactions* | *OR* | *95% CI* | *p-value* |
| Mean of parental loneliness |  |  |  |
| * subjective socioeconomic status | 0.91 | 0.77 - 1.08 | 0.27 |
| * sociability temperament | 1.45 | 0.91 - 2.32 | 0.11 |
| * cognitive performance | 1.06 | 0.73 - 1.55 | 0.75 |
| * depressive symptoms | 1.05 | 0.98 - 1.12 | 0.21 |
| * anxiety symptoms | 2.27 | 0.78 -6.60 | 0.13 |
| Mothers’ loneliness |  |  |  |
| * subjective socioeconomic status | 0.97 | 0.82 - 1.15 | 0.74 |
| * sociability temperament | 1.50 | 0.95 - 2.38 | 0.08 |
| * cognitive performance | 1.02 | 0.67 - 1.56 | 0.92 |
| * depressive symptoms | 1.03 | 0.97 - 1.10 | 0.35 |
| * anxiety symptoms | 1.87 | 0.67 -5.25 | 0.23 |
| Fathers’ loneliness |  |  |  |
| * subjective socioeconomic status | 0.86 | 0.68 - 1.07 | 0.18 |
| * sociability temperament | 1.57 | 0.83 - 2.98 | 0.17 |
| * cognitive performance | 0.96 | 0.6 - 1.52 | 0.85 |
| * depressive symptoms | 1.03 | 0.94 - 1.12 | 0.57 |
| * anxiety symptoms | 2,88 | 0.69 -11.95 | 0.15 |

Note: Adjusted for (in addition to main effects) age (G0 and G1) , sex (G1), education (G1) and income (G1).

| **STable 6. Sensitivity analysis of mediation analysis of parental and offspring loneliness.** | | | | | | | | | | | | | |
| --- | --- | --- | --- | --- | --- | --- | --- | --- | --- | --- | --- | --- | --- |
|  | *Parental loneliness* | | | | | *Mothers loneliness* | | | | *Fathers loneliness* | | | |
| *Mediator* | *Effect* | *RR* | *95% CI* | *E-value* | *95% CI* | *RR* | *95% CI* | *E-value* | *95% CI* | *RR* | *95% CI* | *E-value* | *95% CI* |
| Subjective socioeconmic position |  |  |  |  |  |  |  |  |  |  |  |  |  |
|  | CDE | 1.69 | 1.2-2.36 | 2.76 | 1.7- | 1.57 | 1.11-2.23 | 2.53 | 1.47- | 1.48 | 0.96-2.28 | 2.32 | 1- |
|  | PNDE | 1.69 | 1.2-2.36 | 2.76 | 1.7- | 1.57 | 1.11-2.23 | 2.53 | 1.47- | 1.48 | 0.96-2.28 | 2.32 | 1- |
|  | TNDE | 1.69 | 1.2-2.36 | 2.76 | 1.7- | 1.57 | 1.11-2.23 | 2.53 | 1.47- | 1.48 | 0.96-2.28 | 2.32 | 1- |
|  | PNIE | 1.04 | 0.98-1.11 | 1.25 | 1- | 1.05 | 0.99-1.12 | 1.29 | 1- | 1.02 | 0.94-1.1 | 1.14 | 1- |
|  | TNIE | 1.04 | 0.98-1.11 | 1.25 | 1- | 1.05 | 0.99-1.12 | 1.29 | 1- | 1.02 | 0.94-1.1 | 1.14 | 1- |
|  | TE | 1.76 | 1.25-2.48 | 2.91 | 1.8- | 1.66 | 1.17-2.36 | 2.71 | 1.61- | 1.5 | 0.97-2.33 | 2.37 | 1- |
| Social temperament |  |  |  |  |  |  |  |  |  |  |  |  |  |
|  | CDE | 1.68 | 1.19-2.38 | 2.76 | 1.67- | 1.6 | 1.13-2.26 | 2.58 | 1.52- | 1.54 | 0.99-2.38 | 2.45 | 1- |
|  | PNDE | 1.68 | 1.19-2.38 | 2.76 | 1.67- | 1.6 | 1.13-2.26 | 2.58 | 1.52- | 1.54 | 0.99-2.38 | 2.45 | 1- |
|  | TNDE | 1.68 | 1.19-2.38 | 2.76 | 1.67- | 1.6 | 1.13-2.26 | 2.58 | 1.52- | 1.54 | 0.99-2.38 | 2.45 | 1- |
|  | PNIE | 1.04 | 0.97-1.11 | 1.23 | 1- | 1.05 | 0.98-1.12 | 1.27 | 1- | 0.98 | 0.9-1.07 | 1.17 | -1 |
|  | TNIE | 1.04 | 0.97-1.11 | 1.23 | 1- | 1.05 | 0.98-1.12 | 1.27 | 1- | 0.98 | 0.9-1.07 | 1.17 | -1 |
|  | TE | 1.75 | 1.23-2.49 | 2.89 | 1.76- | 1.68 | 1.18-2.38 | 2.74 | 1.64- | 1.5 | 0.96-2.35 | 2.38 | 1- |
| Cognitive performance |  |  |  |  |  |  |  |  |  |  |  |  |  |
|  | CDE | 1.78 | 1.27-2.48 | 2.95 | 1.85- | 1.61 | 1.14-2.26 | 2.6 | 1.55- | 1.38 | 0.9-2.11 | 2.09 | 1- |
|  | PNDE | 1.78 | 1.27-2.48 | 2.95 | 1.85- | 1.61 | 1.14-2.26 | 2.6 | 1.55- | 1.38 | 0.9-2.11 | 2.09 | 1- |
|  | TNDE | 1.78 | 1.27-2.48 | 2.95 | 1.85- | 1.61 | 1.14-2.26 | 2.6 | 1.55- | 1.38 | 0.9-2.11 | 2.09 | 1- |
|  | PNIE | 1.01 | 0.98-1.04 | 1.12 | 1- | 1 | 0.98-1.03 | 1.06 | 1- | 1.04 | 0.98-1.1 | 1.24 | 1- |
|  | TNIE | 1.01 | 0.98-1.04 | 1.12 | 1- | 1 | 0.98-1.03 | 1.06 | 1- | 1.04 | 0.98-1.1 | 1.24 | 1- |
|  | TE | 1.8 | 1.28-2.52 | 2.99 | 1.88- | 1.61 | 1.15-2.27 | 2.61 | 1.56- | 1.43 | 0.93-2.19 | 2.21 | 1- |
| Depressive symptoms |  |  |  |  |  |  |  |  |  |  |  |  |  |
|  | CDE | 1.56 | 1.08-2.24 | 2.48 | 1.37- | 1.32 | 0.91-1.91 | 1.98 | 1- | 1.4 | 0.89-2.19 | 2.14 | 1- |
|  | PNDE | 1.56 | 1.08-2.24 | 2.48 | 1.37- | 1.32 | 0.91-1.91 | 1.98 | 1- | 1.4 | 0.89-2.19 | 2.14 | 1- |
|  | TNDE | 1.56 | 1.08-2.24 | 2.48 | 1.37- | 1.32 | 0.91-1.91 | 1.98 | 1- | 1.4 | 0.89-2.19 | 2.14 | 1- |
|  | PNIE | 1.21 | 1.03-1.42 | 1.72 | 1.22- | 1.29 | 1.11-1.5 | 1.9 | 1.45- | 1.09 | 0.94-1.26 | 1.41 | 1- |
|  | TNIE | 1.21 | 1.03-1.42 | 1.72 | 1.22- | 1.29 | 1.11-1.5 | 1.9 | 1.45- | 1.09 | 0.94-1.26 | 1.41 | 1- |
|  | TE | 1.89 | 1.28-2.79 | 3.18 | 1.87- | 1.7 | 1.15-2.53 | 2.8 | 1.56- | 1.52 | 0.95-2.45 | 2.42 | 1- |
| Anxiety symptoms |  |  |  |  |  |  |  |  |  |  |  |  |  |
|  | CDE | 1.55 | 1.08-2.21 | 2.46 | 1.38- | 1.55 | 1.08-2.21 | 2.46 | 1.38- | 1.39 | 0.89-2.17 | 2.12 | 1- |
|  | PNDE | 1.55 | 1.08-2.21 | 2.46 | 1.38- | 1.55 | 1.08-2.21 | 2.46 | 1.38- | 1.39 | 0.89-2.17 | 2.12 | 1- |
|  | TNDE | 1.55 | 1.08-2.21 | 2.46 | 1.38- | 1.55 | 1.08-2.21 | 2.46 | 1.38- | 1.39 | 0.89-2.17 | 2.12 | 1- |
|  | PNIE | 1.18 | 1.04-1.34 | 1.65 | 1.26- | 1.18 | 1.04-1.34 | 1.65 | 1.26- | 1.09 | 0.95-1.25 | 1.4 | 1- |
|  | TNIE | 1.18 | 1.04-1.34 | 1.65 | 1.26- | 1.18 | 1.04-1.34 | 1.65 | 1.26- | 1.09 | 0.95-1.25 | 1.4 | 1- |
|  | TE | 1.83 | 1.26-2.66 | 3.06 | 1.82- | 1.83 | 1.26-2.66 | 3.06 | 1.82- | 1.51 | 0.95-2.4 | 2.39 | 1- |

CDE = controlled direct effect; PNDE = pure natural direct effect; TNDE= total natural direct effect; PNIE = pure natural indirect effect; TNIE= total natural indirect effect; TE = total effect
